# Repair of an inguinoscrotal hernia containing the urinary bladder: a case report

**DOI:** 10.1186/1752-1947-6-90

**Published:** 2012-03-26

**Authors:** Georgios I Panagiotakis, Konstantinos G Spyridakis, Maria N Chatziioannou, Nikolaos G Kontopodis, Stylianos E Kandylakis

**Affiliations:** 1 1st Surgical Department, Venizelio General Hospital, Knossos Avenue, M.B. 44 Heraklion, Greece; 2 Radiology Department, Venizelio General Hospital, Knossos Avenue, M.B. 44 Heraklion, Greece

## Abstract

**Introduction:**

Cases of patients with inguinoscrotal hernia containing the urinary bladder are very rare. These patients usually present with frequent episodes of urinary tract infection, difficulty in walking, pollakisuria and difficulty in initiating micturition because of incarceration of the urinary bladder into the scrotum.

**Case presentation:**

We describe the case of an 80-year-old Caucasian man with an incarcerated urinary bladder into the scrotum who underwent surgical repair with mesh.

**Conclusions:**

Diagnosis of such cases often requires not only clinical examination but also specialized radiological examinations to show the ectopic position of the urinary bladder. Surgical repair in these patients is a real challenge for surgeons.

## Introduction

Inguinoscrotal hernia containing the urinary bladder is a highly rare condition, and it is always associated with recurrent episodes of urinary tract infection (UTI). To the best of our knowledge, only a few cases have been reported in the literature to date. We report the case of an 80-year-old Caucasian man who presented to our Emergency Department (ED) with fever and a right direct inguinoscrotal hernia containing the urinary bladder. The patient underwent surgical repair of the hernia with mesh and restoration of the urinary bladder to its normal physical position. Right orchiectomy was also performed. He was discharged in good condition on the sixth post-operative day. Surgical repair of an inguinoscrotal hernia containing the urinary bladder can be achieved with satisfactory results after proper pre-operative preparation of patients.

## Case presentation

**Figure 1:**
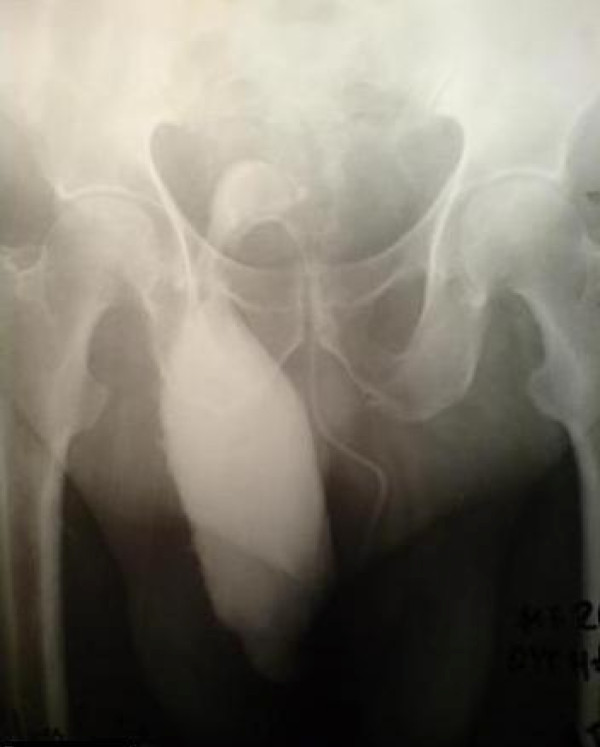
**Pre-operative contrast-enhanced retrograde cystography**. The incarcerated urinary bladder into the scrotum is shown.

**Figure 2:**
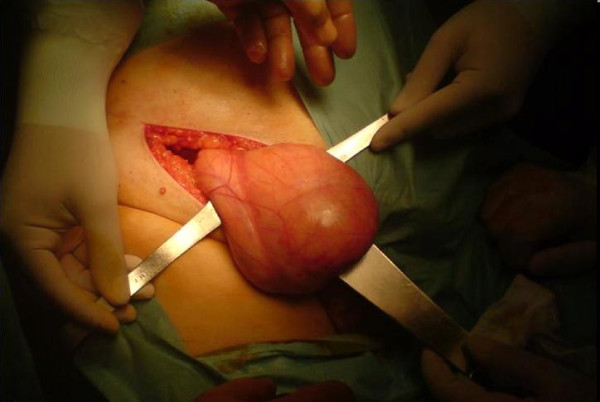
**The urinary bladder after the adhesiolysis at the bottom of the hernia sac**.

**Figure 3:**
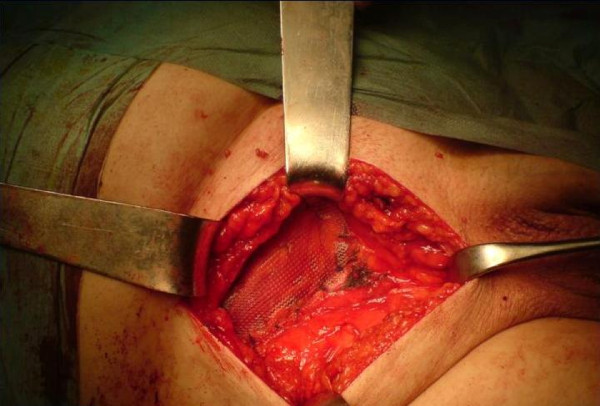
**The repair of the direct hernia with the mesh**.

**Figure 4:**
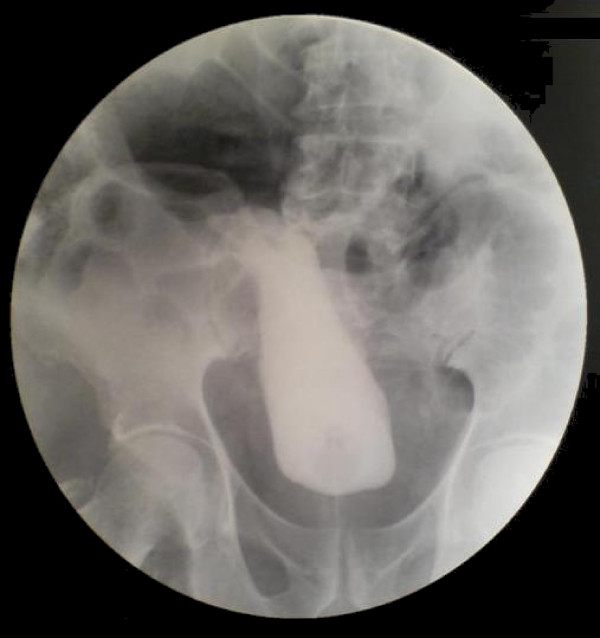
**Post-operative contrast-enhanced retrograde cystography**. The procedure was performed again to ensure the induction of the urinary bladder to its normal position.

An 80-year-old Caucasian man presented to the ED with high fever, strangury, difficulty in initiating micturition and walking during the previous 6 days. His relevant medical history included chronic obstructive pulmonary disease, arterial hypertension, type 2 diabetes mellitus and coronary heart disease, all of which were controlled with medication. He also mentioned recurrent episodes of UTI. The clinical examination revealed a right direct inguinoscrotal hernia, and contrast-enhanced retrograde cystography (CERC) proved that the urinary bladder was incarcerated into the scrotum (Figure 1). The testicles were impalpable. After adequate pre-operative preparation, the patient underwent surgical repair of his hernia. Antibiotics for a diagnosed UTI were given to the patient at the time of his admission to the surgical department. Pre-operatively, a nasogastric tube and Foley catheter were also inserted. The hernia was initially approached through a transverse groin incision. Severe adhesions were anticipated because of the chronic nature of the hernia (Figures 2 and 3). Indeed, during adhesiolysis at the bottom of the hernial sac, the right testicle appeared to be atrophic because it was surrounded by multiple severe inflammatory adhesions, necessitating right-sided orchiectomy. The bladder was pushed back to its normal anatomic position, thus protecting its innervation and the right ureter. A hernioplasty was performed with a polypropylene pre-fabricated one-lay mesh (Prolene; Ethicon, Somerville, NJ, USA), which was riveted in the pre-peritoneal space, using the Lichtenstein open tension-free hernioplasty technique. Right after the operation a new CERC was performed to ensure the success of the operation (Figure 4). The patient remained under close post-operative medical observation and antibiotic treatment for 10 more days without complications. He was discharged in optimal condition on the sixth post-operative day.

## Discussion

Inguinoscrotal hernias containing the urinary bladder are very rare, and their repair is a real challenge for surgeons [1,2]. They are uncommon in developed countries, and patients with such problems usually present with frequent UTI after years or even decades of neglect [3]. Generally, the UTIs are of a recurrent nature, and, apart from the classical complications of inguinal hernias, the specific problems cause dramatic impairment of the patient's quality of life [4]. The mobility of these patients is accordingly restricted, and they often have recurrent episodes of UTI for which they should receive appropriate antibiotic treatment [5,6]. Giant inguinoscrotal hernias are also often associated with extreme visceroptosis and tissue expansion of vascular pedicles [7,8]. In cases that raise clinical suspicion of incarcerated viscera into an inguinoscrotal hernia, radiology can play an important role in facilitating the diagnosis.

Minordi *et al. *[1] reported a case of a patient who presented with a massive inguinoscrotal vesical hernia complicated by bladder rupture, which was pre-operatively diagnosed on the basis of sonography and computed tomographic cystography. It is often necessary to execute adequate radiological examinations pre-operatively to prove the grade of incarceration of the bladder into the scrotum. In our present case, the contrast-enhanced retrograde cystography which was performed pre-operatively helped us not only to diagnose the problem but also to decide on the suitable surgical technique to be performed. The same radiological examination performed post-operatively proved the appropriate induction of the urinary bladder back to its normal anatomic position. Giant inguinoscrotal hernias often result in compromise of respiratory and cardiac function because of the augmentation of the intra-abdominal pressure that can cause abdominal compartment syndrome [9]. These kinds of problems can be present in cases that involve repair of giant inguinoscrotal hernias containing parts of the small or large intestine when the viscera is induced into the abdominal cavity [3,10,11]. Fortunately, this was not the case in our patient, as we only induced the urinary bladder into its anatomic position, which does not cause abdominal compartment syndrome.

Moreover, the choice of the right surgical technique is crucial. The Lichtenstein open tension-free hernioplasty technique that we used seems to be the best option in such patients. The mesh is positioned in the pre-peritoneal space. The patch decreases the tension on the weakened abdominal wall, thus reducing the risk of hernia recurrence. Serious problems can emerge in cases of giant inguinoscrotal hernias when the herniated viscera are induced *de novo *into the abdominal cavity [12]. A surgeon should always bear in mind that if a massive hernia is induced abruptly into a contracted peritoneal cavity, the patient might run the risk for a sudden increase in intraabdominal and intra-thoracic pressure, which might precipitate fatal cardiorespiratory failure [6,10,13]. Moreover, it should be noted that post-operative ileum could further increase intra-abdominal and intra-thoracic pressure, and, in parallel, reduction of a massive hernia under excessive tension is associated with a high incidence of wound dehiscence and recurrence of the hernia [2,14,15].

There are also other important complications that can emerge after the surgical repair of an inguinoscrotal hernia. Patients who undergo hernia repair are at risk for reaction to anesthesia (the main risk), site infection and bleeding, nerve damage, numbness of the skin and loss of blood supply to the scrotum or testicles which results in testicular atrophy (all of the latter risks being infrequent). These patients can also present with post-operative complications, such as damage to the cord that carries sperm from the testicles to the penis (vas deferens), resulting in inability to father children or even damage to the femoral artery or vein. Fortunately, in our patient, we did not face any kind of post-operative complications, and the patient's recovery was uneventful.

The repair of an inguinoscrotal hernia containing the urinary bladder is a great challenge for surgeons. The correct pre-operative management, the appropriate radiological examinations and the right surgical method chosen by the surgeon can all lead to a successful result. Surgical therapy, though challenging and demanding, may be the only mode of treatment that can offer these patients a return to a satisfactory level of function and quality of life. Informed consensus is always necessary and ensures that the patient is fully aware of all the pros and cons of the possible post-operative complications.

## Conclusions

This brief report highlights the potential risk that can occur in cases of an incarcerated urinary bladder into the scrotum. The clinical examination and contrast-enhanced retrograde cystography, a low-cost technique, can be performed to confirm the diagnosis. Surgical repair is the correct treatment choice for these patients.

## Consent

Written informed consent was obtained from the patient for publication of this case report and any accompanying images. A copy of the written consent is available for review by the Editor-in-Chief of this journal.

## Abbreviations

CERC: contrast-enhanced retrograde cystography; CT: computed tomography; ED: emergency department; UTI: urinary tract infection.

## Competing interests

The authors declare that they have no competing interests.

## Authors' contributions

GIP and KGS analyzed and interpreted the patient data and were the major contributors to the writing of the manuscript. MNC performed the radiology examination. SEK, GIP and NGK performed the surgical treatment. All authors read and approved the final manuscript.
